# Investigating the average kiloelectron-volt emission of partially observed events in nuclear physics through distance weighted mean based censored control chart

**DOI:** 10.1371/journal.pone.0308822

**Published:** 2024-11-05

**Authors:** Shumaila Nisar, Syed Muhammad Muslim Raza, Olayan Albalawi, Aiedh Mrisi Alharthi

**Affiliations:** 1 Department of Economics and Quantitative Methods, Dr Hasan Murad School of Management, University of Management and Technology, Lahore, Pakistan; 2 Department of Statistics, Faculty of Science, University of Tabuk, Tabuk, Saudi Arabia; 3 Department of Mathematics, Turabah University College, Taif University, Taif, Saudi Arabia; University of Hamburg: Universitat Hamburg, GERMANY

## Abstract

The average lifespan of particles, a crucial parameter in nuclear physics, is essential for identification purposes. Modern particle detectors excel at recognizing individual radioactive nuclei arrivals and their subsequent decay events. However, challenges arise when matching arrivals with departures, especially when departures are only partially observed. One inefficient approach involves conducting experiments with very low arrival rates to facilitate matching. The kiloelectron-volt E(keV) emission is obtained during this radio active process. This study focuses on the meticulous surveillance of the average keV emission from partially observed events within the domain of nuclear physics. To accomplish this, the methodology employs the statistical approach known as Distance Weighted Mean (DWM), integrated with the application of censored control charts. The utilization of censored control charts allows for the effective management of incomplete data, enabling researchers to make informed decisions despite potential limitations in observation. We propose a DWM based exponentially weighted moving average-cumulative sum (DWM-EC) control chart for monitoring kiloelectron-volt E(keV) data. The proposed charts is developed for Weibull lifetimes under type-I censoring. For the construction of an efficient control charting structure, we employed the conditional median (CM) methods. The goal is to find changes in the mean of Weibull lifetimes with censored data with known and estimated parameter conditions. The performance of the proposed DWM-EC chart is evaluated by the average run length (*ARL*). Besides a simulation study, a real-life data set on E(keV) related to the alpha decays of 177 Lutetium isotope is also discussed.

## 1. Introduction

A classic example of a Poisson process over time is radioactive decay. The physics behind this statistical framework is as follows: lives follow an exponential distribution with a mean equal to the inverse of the decay rate, and decay events are independent as long as the decay rate is known. In nuclear physics, the mean lifespan is crucial because it accurately captures the structure of the underlying quantum mechanical states. Notably, radioactive alpha decays longer than those anticipated by a simple model can result from significant changes in nuclear structure between beginning and final states [1]. In particle physics, estimating the mean lifespan under different experimental configurations is a well-known statistical issue [2].

Modern experiments are able to manufacture radioactive species on a continuous basis and record their decays concurrently because of sophisticated particle detectors and data collecting systems that can distinguish between the arrival of single radioactive nuclei and their subsequent decays [3, 4]. For the sake of generality, these arrivals and decays are referred to as departures since they frequently stay mismatched, making it impossible to directly link an arrival to a departure. A specific arrival and departure pair may only be identified with confidence when low arrival rates and short mean lifetimes are present, as evidenced by the observation of just one departure between successive arrivals.

When certain arrivals or departures are mislabeled or only partially noticed, problems might occur (censored). This work focuses on studies when structural restrictions of the particle detector prevent the collection of all departures. For example, when alpha radioactive nuclei produced in fusion events are implanted onto a detector, they are usually placed in close proximity to the detector’s surface. As a result, around half of the departures are missed because the alpha particle escapes the detector. This results in an uneven number of arrival and departure times that cannot be consistently connected, along with the problem of matching arrivals with departures. The challenge of determining the mean lifetime and keeping track of such data using the suggested censored control charts is discussed in the study situations.

In statistical process control (SPC), control charts are an effective tool for tracking and analyzing operations across time. They support businesses in making ensuring that their procedures are reliable, consistent, and kept within predetermined bounds. Control charts make it possible to spot patterns and variances that could point to possible problems or process enhancements. Control charts are widely used in many different sectors and are essential for process optimization and quality control.

In the manufacturing industry, control charts are widely utilized to oversee and regulate production procedures, guaranteeing uniform product quality and reducing errors. In the medical field, control charts are used to track medical mistakes, keep an eye on patient outcomes, and pinpoint areas where procedures may be improved. They are used in the finance industry to track important financial indicators and identify patterns and abnormalities, such as transaction processing times, mistake rates, and stock price swings. In the service business, control charts are used to track customer satisfaction ratings, process efficiency, and service delivery parameters. In order to assist teams improve software quality and delivery, control charts are used in software development to track errors, code reviews, and development cycle duration. Control charts are used in environmental monitoring to monitor pollution levels, other environmental indicators, and the quality of the air and water. SPC charts are a useful tool for tracking and enhancing supply chain operations, including order fulfillment, inventory control, and delivery schedules.

Control charts enable organizations to keep an ongoing eye on a process to make sure it’s functioning within reasonable bounds. They aid in separating variations with common causes—those innate to the process—from variations with unique causes—those brought on by outside influences or particular occurrences.

To ensure the accuracy and dependability of the analysis while using control charts, it is crucial to collect enough representative data and to sample properly. Furthermore, control charts are only one component of a larger statistical process control system that also consists of methods for process data gathering, and analysis.

Partially or fully censored data are frequently encountered in reliability engineering and medical research, two domains where filtered data is highly relevant. When an observation’s true value is unknown due to a restricted measurement or a continuing observation at the conclusion of the research period, this is known as censoring. There are several different ways that data may be censored, including progressive, Type-II, interval, and Type-I censoring. The kind of statistical analysis that may be performed on the data depends on the censoring method. Erroneous conclusions and biased estimations might arise from disregarding censored data or interpreting it as missing. As a result, it’s critical to create statistical techniques that can effectively handle censored data. As was previously mentioned, one technique for keeping an eye on data that has been suppressed is the use of control charts. Researchers can increase their grasp of the underlying phenomena they are studying and acquire more precise estimations of parameters by taking into consideration censored data. Better decision-making and better product or process design may result from this, and these developments may ultimately have a big influence on a lot of different sectors and industries, like engineering, manufacturing, healthcare, and medicine.

Finding assignable causes in lifetime data is a difficult yet fascinating undertaking, particularly in industrial and medical research. Nevertheless, inadequate failure-time information—also referred to as filtered data is produced by time and budget constraints. The performance of traditional control charts, such as Shewhart charts, to track trials for potential assignable reasons for process improvement is significantly lower than that of censored control charting based on CEV. New control charting techniques were suggested by [5, 6] for handling entire data. When it comes to handling filtered data, traditional charts typically do not respond quickly enough, which limits their ability to discriminate. presented a one-side chart utilizing the conditional expected value (CEV) in order to get around these unfavourable aspects of the monitoring techniques for censored data. Through an empirical analysis, the authors demonstrated how the concept enables fast process degradation identification for heavily filtered data monitoring. [7] proposed Shewhart control charts using the CEV approach; they were later shown to work well for heavily filtered data in industrial and medical applications. [8] suggested EWMA control charts with lower and upper sides based on the CEV method to identify changes in the Weibull quality attributes mean. Similarly, to monitor type-I censored data assuming the gamma and Gompertz distributions, respectively [9, 10], presented CEV EWMA charts. Recent advancements in control charts for data monitoring in real-world applications are evidenced in references [11–18].

Control charts were also introduced [19, 20] to monitor type-I censored data, adhering to the CEV method. Because most lifespan distributions are skewed, the CEV technique might not be applicable, it should be highlighted. In contrast to the current methods [21], suggested a Shewhart chart based on conditional median (CM). In a simulated analysis, the authors [22–26] demonstrated that their hybrid EWMA control chart with repeated sampling works better than CEV charts. This study focuses on type-I censoring, which is often used in industry, even though monitoring methods for type-II censored data have been reported in the literature. Differentiating between the CM, imputation, and CEV approaches is crucial. From a methodological standpoint, all three strategies replace missing or censored data in order to improve the estimate. Imputation techniques are employed to replace missing observations in the data, while the conditional mean and conditional median are used to replace censored observations in the CEV and CM, respectively.

This study introduces the previously unexplored in the literature Distance Weighted Mean—exponentially weighted moving average-cumulative sum (DWM-EC) control chart based on CM. Because of its adaptability and usefulness in reliability engineering and nuclear physics, the Weibull distribution’s mean level is monitored in this study with an emphasis on type-I censored data. However, the suggested method may be expanded to include other reasonable distributions of life expectancy. When it comes to assignable causes, the innovative charts perform better than traditional CM-based EWMA charts. The study also examines the performance of the DWM-EC chart when the maximum likelihood estimation technique is used to estimate the scale parameter. The effectiveness of the recommended charts is evaluated using the average run length (ARL).

Additionally, this page provides a comparison between the censored DWM-EC charts with the censored EWMA and CM-CUSUM charts.

The study’s remaining sections are arranged as follows: Section 2 displays the CM’s derivations. In addition to parameter estimates, Section 2 provides the censored DWM-EC’s design structure. In Section 3, it is explained how the censored DWM-EC, EWMA, and CUSUM charts performed at various censoring rates. To demonstrate the suggested technique, a dataset on the reaction time of an experiment using electric sockets is shown in Section 4. Section 5 has closing thoughts.

## 2. The cm based DWM-EC control charts

This section introduces the Conditional Median based Distance Weighted Mean—exponentially weighted moving average-cumulative sum (CM-DWM-EC) charts to monitor the mean of the Weibull distribution, that is, the variable of interest *X* denotes the lifetime of a product that is assumed to follow a Weibull distribution. The Weibull distribution is the most commonly used probability distribution in reliability analysis, engineering, and medical studies. The probability density function of a Weibull random variable *X* is given by:

$$f(x,\alpha ,\beta )=\frac{\beta }{\alpha^{\beta }}x^{\beta -1}\exp(-{\left[x/\alpha \right]}^{\beta })\;x>0$$ (1)

where *α* is the scale parameter and *β* is the shape parameter, respectively.

Lets denote *X*_*i1*_, *X*_*i2*_ *…*.. *X*_*in*_ the actual lifetime while *T*_*i1*_, *T*_*i2*_ *…*.. *T*_*in*_, *i = 1*, *2…*, *δ* denote the lifetimes of failed units in a life testing experiment, i.e., obtained after exercising the type-I right censoring mechanism. Here, δ denotes the subgroup size, which may be variable depending upon the situation. The *r* is random here while *n* and C (censoring time ‘C’) are fixed in advance.

Then, we compute the censoring rate by *P*_*c*_ *= 1-F(x = c; α*,*β)*, where *F(x; α*,*β)* is the cumulative density function of the Weibull distribution, that is, *P*(*X*≤*C*) = 1−exp(−[C/*α*]^*β*^). The mean is denoted by *μ* and is given as:$\mu =E(x)=\int_{0}^{\infty }xf(x)dx=\alpha \Gamma (1+\frac{1}{\beta })$.

The Conditional Median is expressed as:

$$m={\left[-{\alpha_{0}}^{\beta }\ln(\frac{2-\exp(-D_{c})}{2})\right]}^{1/\beta_{0}}$$ (2)

where $D_{c}={\left(C/\alpha_{0}\right)}^{\beta_{0}}$, lower incomplete gamma function $\Gamma (x,a)=\int_{y=0}^{x}y^{a-1}\exp(-y)dy$, and *α*_0_, *β*_0_ are the stable-process values of *α* and *β*, respectively.

Further to this point, we will fix the shape parameter and focus on the scale parameter [8], i.e., to calculate the CM, we suppose that the scale parameter is fixed and known. However, in practice, we often estimate unknown parameters from the *Phase-I* dataset. For the estimation of the unknown scale parameter, the method of maximum likelihood estimation (MLE) is discussed in the next section. After calculating the CM we replace the Censored observations by CM and use the transformed data to calculate the Distance Weighted Mean using the formula given below:

Let the observations y_1,_ y_2,_ ……………..y_n_ shows the transformed data with weights w_1_,w_2_,…………..w_n_

$$T_{DWM}=\frac{\sum_{i=1}^{n}w_{i}y_{i}}{\sum_{i=1}^{n}w_{i}}$$ (3)

The weighting coefficient for Y_i_ is computed as the inverse mean distance between y_i_ and the other data points.


$$w_{i}=\frac{k}{\left|y_{i}-y_{j}\right|}$$ (4)

Where k is a positive quantity. In literature mostly it is taken as 1 or n-1.

### 2.1 Estimation of *α*

To estimate the unknown scale parameter, we first write the likelihood function. The MLE under type-I censoring is given as ${\hat{\alpha }}_{MLE}=\frac{1}{r}{\left[\sum_{i=1}^{r}\phi_{i}{\left(X_{i}\right)}^{\beta_{0}}+(n-k)C^{\beta_{0}}\right]}^{\frac{1}{\beta_{0}}}$, where *r* represents the censored units per subgroup, *n* represents the sample size, *X*_*i*_ (i = 1,2,3,…,n) shows the lifetime from the Weibull distribution [11].

### 2.2 CM based DWM-EC control charts

To define DWM-EC control chart, assume *λ*_1_,*λ*_2_,*λ*_3_∈[0,1], the CM-DWM-EC statistic in a relative form can be defined as follows:

$$HDWCM_{(cm)i}=max\{HDWCM_{i-1}+\bar{DWC_{i}^{CM}}-m_{o},m_{o}\}/min\{HDWCM_{i-1}+\bar{DWC_{i}^{CM}}-m_{o},m_{o}\},i=1,2,3,\ldots ,\delta$$ (5)

The quantity *m*_*o*_ in Eq (5) is a barrier and used to increase the sensitivity of the CM Based DWM-EC *c*ontrol chart. Thus, it needs to be chosen carefully and a very natural choice is *m*_*o*_ = $\alpha_{0}\Gamma (1+\frac{1}{\beta_{0}})$. However, the starting value *HDWCM*_0_ is assumed zero in this study. The *HDWCM* is the EWMA based test statistics calculated using the DWM estimator. As discussed previously, for both upward and downward shifts in the process mean, the *HDWCM*_*i*_ statistic increases (Eq 5), and hence only the upper control limit is required to detect out-of-control signals. Let the upper limit is denoted by *UCL*_*HDWCM*(*CM*)_ for the CM charts, respectively. Furthermore, the control limits will always be greater than one, as the minimum value is in the denominator of Eq.3.

The *ARL* is the most widely practiced performance assessment criterion and the *ARL* computed from the in-control data is known as the in-control *ARL* and denoted by *ARL*_*o*_, while the *ARL* calculated from *Phase-II* data (that is, from a shifted process) is known as the out-of-control *ARL* and denoted by *ARL*_*1*_. In general, the *ARL* is the average number of points plotted on a chart before a special cause signal is raised. Generally, *ARL*_*o*_ is fixed large while a chart with a smaller *ARL*_*1*_ is said to be more efficient than the chart having a larger *ARL*_*1*_.

## 3. Performance evaluations

The efficiency of the CM-DWM-EC charts is discussed in this section. Besides this, a comparison of CM-DWM-EC charts to the CM based EWMA and CM based CUSUM charts is also given in this section.

To investigate the efficiency of the charts, the Monte Carlo simulation approach is used to calculate the *ARL*. The *ARL* assessment of the *CM-DWM-EC* charts is discussed assuming known and estimated scale parameter cases while keeping fixed the shape parameter.

### 3.1 Stepwise algorithm for assessing CM-DWM-EC chart performance

For assessing the performance of CM-DWM-EC charts, we fixed the desired *ARL*_*0*_ and find the corresponding *UCL* values, respectively, for the given *δ* and *n*. The steps to determine the control limits and ARLs for the pre-fixed values of Pc, mo, *n* and *ARL*_*0*_ are given below:

1. Fix P_c_ (Censoring rate) and subgroup size. Use the Phase-I data set to estimate *α* if it is unknown.
2. Substitute the censored observations with their Censored median (CM).
3. Now Compute the DWM statistic data for different subgroups.
4. Compute the CM-DWM-EC statistics data based on Eq 5.
5. Then, calculate the (1−*p*)−*th* quantile point, where *p* is pre-specified false alarm probability.
6. Repeat the above step L-times (e.g., 100000 times) and compute the average to have the UCLs of the CM-DWM-EC chart.
7. Continue to plot the value of the CM-DWM-EC statistic against the subgroup numbers until the test statistic crosses the control limit. Record the corresponding subgroup number at which the first out-of-control signal appears.
8. Repeat Step 4 say M times (e.g., 100000 times) and compute the average, which is the *ARL*_0_.

To calculate the out-of-control *ARL*, generate data from the Weibull distribution with the shifted parameter and check it against the monitoring threshold in Step 3. Store the subgroup numbers at which the monitoring statistic first falls beyond the control limit. Repeat this step many times and compute the average, which is *ARL*_1_.

#### Effect of estimation

From Table 2: The findings reveal significant insights into the performance of different control charts under various conditions. Specifically, comparing *ARL*_1_ values for different shifts and censoring rates, it’s evident that the CM-CUSUM chart outperforms the CM-EWMA chart for higher shift i.e 30% increase in shifts. Furthermore, the impact of parameter estimation on the CM-DWM-EC chart is noteworthy, with *ARL*_1_ values being smaller when parameters are known compared to when they are estimated. Additionally, analyzing the *ARL*_1_ values for different shifts and censoring rates with the CM-DWM-EC chart demonstrates a clear trend: for increasing shifts, *ARL*_1_ values decrease as censoring rates increase, and vice versa for decreasing shifts. However, the superiority of the CM-DWM-EC chart remains consistent across various combinations of shifts and censoring rates, indicating its robustness. These findings underscore the effectiveness of the CM-CUSUM chart over the CM-EWMA chart and highlight the impact of parameter estimation on the CM-DWM-EC chart. Moreover, the consistent superiority of the.

From the Tables 1–3 it is also observed that as the censoring rate increases from 20% to 40% for a 30% increase in shift, the *ARL*_*1*_ values rise, indicating lack in performance. Conversely, for a 20% to 40% shift at the 30% decrease in shift level, the *ARL*_*1*_ values decrease, demonstrating that the chart’s efficiency improves with the increase in censoring rates for a specified decrease shift level.

**Table 1:** Out-of-control *ARL* values for CM-CUSUM, CM-EWMA and CM-DWM-EC control chart sequences *for n = 3 and $ARL_{0}=100,\alpha =0.5,\beta =0.5,\lambda_{1}=\lambda_{2}=0.2,\lambda_{3}=0.3.$*.

*n*	3											
* *P_c_	CM-EWMA CHART				CM-CUSUM CHART				CM-DWM-EC CHART			
	Shifts				Shifts				Shifts			
	30% increase	30% decrease	20% increase	20% decrease	30% increase	30% decrease	20% increase	20% ecrease	30% increase	30%decrease	20% increase	20% decrease
0.2	6.72	4.49	9.09	13.55	6.27	2.85	9.12	11.36	5.64	2.71	6.63	10.16
0.3	10.67	2.73	19.49	9.93	9.29	1.46	17.77	10.02	8.02	2.2	15.40	9.54
0.4	17.67	1.35	44.69	6.93	17.28	1	43.37	5.20	16.08	1.30	42.70	3.33

**Table 2:** Out-of-control *ARL* values for CM-CUSUM, CM-EWMA and CM-DWM-EC control chart sequences *for n = 3 with MLE*. $\overset{⌢}{\alpha }=0.475,\beta =0.5,ARL_{0}=100,\lambda_{1}=\lambda_{2}=0.2,\lambda_{3}=0.3.$.

*n*	3											
* *P_c_	CM-EWMA CHART				CM-CUSUM CHART				CM-DWM-EC CHART			
	Shifts				Shifts				Shifts			
	30% increase	30% decrease	20% increase	20% decrease	30% increase	30% decrease	20% increase	20% decrease	30% increase	30% decrease	20% increase	20% decrease
0.2	10.74	5.46	16.65	14.10	8.98	5.57	10.93	12.94	6.77	4.49	6.22	11.81
0.3	12.25	4.49	27.53	14.13	11.97	4.44	24.50	11.07	9.57	3.41	22.14	9.98
0.4	23.11	4.05	48.78	9.11	22.83	1.03	50.45	7.28	21.28	1.91	47.35	5.09

**Table 3:** Out-of-control *ARL* values for CM-CUSUM, CM-EWMA and CM-DWM-EC control chart sequences *for n = 7 with $\alpha =1,\beta =0.75,ARL_{0}=200,\lambda_{1}=\lambda_{2}=0.2,\lambda_{3}=0.3.$*.

*n*	7											
* *P_c_	CM-EWMA CHART				CM-CUSUM CHART				CM-DWM-EC CHART			
	Shifts				Shifts				Shifts			
	30% increase	30% decrease	20% increase	20% decrease	30% increase	30% decrease	20% increase	20% decrease	30% increase	30% decrease	20% increase	20% decrease
0.2	7.17	4.93	10.55	11.21	6.28	3.67	11.39	10.13	6.36	3.31	9.59	9.68
0.3	11.78	3.12	26.08	10.67	8.74	2.83	22.52	9.38	8.37	2.59	21.48	8.50
0.4	22.74	3.05	46.85	7.61	21.44	1.81	45.54	5.63	21.10	1	44.38	5.37

From Tables 1–3 it is observed that, when the censoring rate is low, the CM-DWM-EC chart detects an increasing shift in the scale parameter more effectively than a decreasing shift. However, when censoring rates are high and shifts are decreasing, the chart’s practicality is not diminished when compared to the CM-CUSUM chart. Overall, for both increasing and decreasing shifts, the CM-based DWM-EC chart outperforms its counterpart. The chart’s average run length (*ARL*) attribute is unbiased, which implies that while the process is under control, the *ARL* never surpasses the *ARL*_0_. The performance of the chart, like those of other charts, is significantly connected with parameter estimate, and a large sample size is advised to overcome this estimating impact and attain the desired results.

## 4. Applications

An illustrative example involving kiloelectron-volt (keV) data is utilized to showcase the proposed control charting methodology. The data, provided in S2 Appendix, is obtained by converting electron-volt (eV) measurements to keV by dividing by 1000. The data distribution is approximated as a Weibull distribution with an unknown scale parameter, estimated using easyfit software.

**Fig 1:**
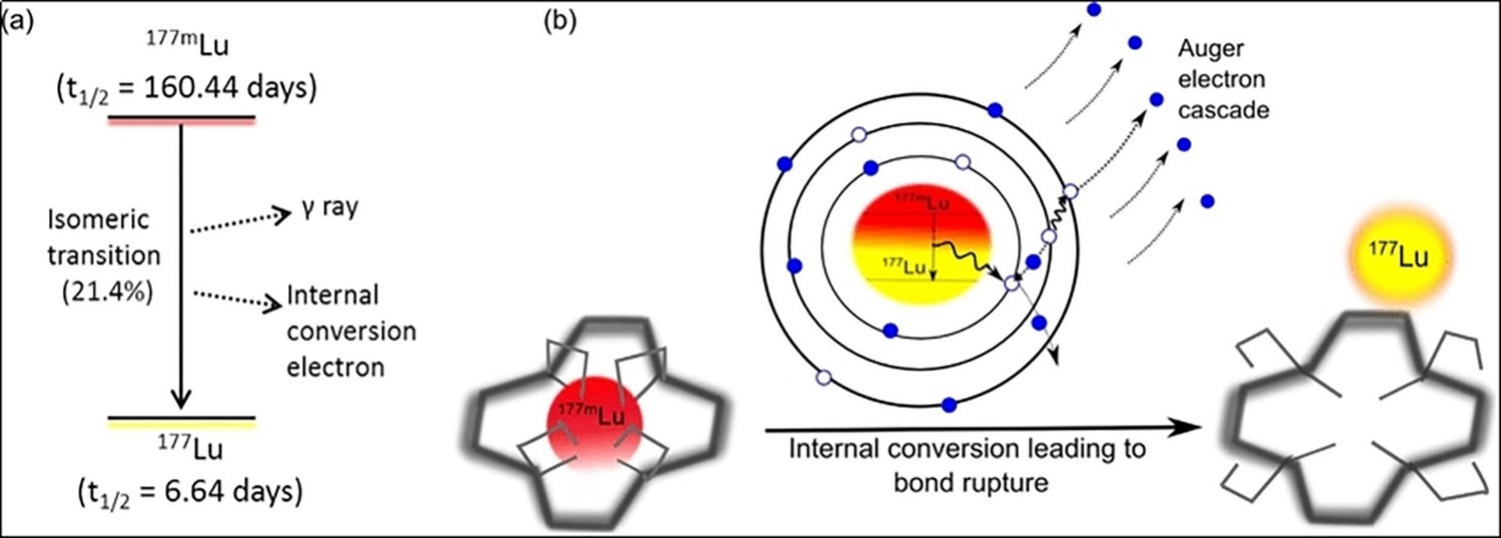
Schematic representation of the decay process. (a) Decay scheme of 177m Lu to 177 Lu. (b) Process of bond rupture.

The Fig 1 represents the metastable isomer 177m Lu is coordinated to a very stable complex (left side). During the decay via internal conversion the nucleus excess of energy is transferred to an inner electron causing an auger electron cascade (center). After the cascade the atom is in a highly charge state, the chemical bonds are broken and the freed 177 Lu can be separated (right side).

By Bootstrapping, selects samples of size 3, and the maximum likelihood estimation method, employing the first 45 observations as phase-I data, determines the estimated scale parameter as 1.85. Assuming a 45% censoring rate and a 30% decrease in mean as the shift to be detected, the proposed hybrid control chart is formulated.

**Fig 2:**
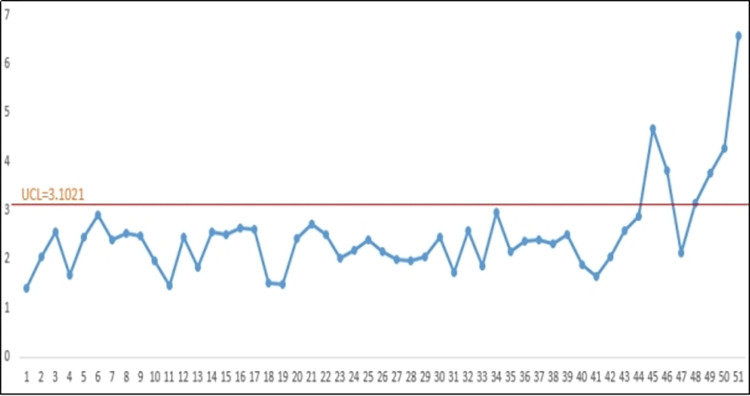
CM-DWM-EC chart using 30% decrease in the mean for the E(KeV).

Fig 2 illustrates that the CM DWM-EC control charts do not signal any out-of-control indications until the 46th sample. To evaluate the efficacy of the proposal, a dataset comprising 20 observations is generated after the 45th subgroup. Introducing a 30% decreasing shift in the mean of the Weibull distribution for the shifted data, with a censoring time of 58.4 and *ARL*_o_ = 47, the CM value is calculated as 15.56. Notably, for the shifted samples, the CM-DWM-EC chart detects an out-of-control signal as early as the 2nd sample (refer to Fig 1).

## 5. Conclusion

This article introduces CM-DWM-EC charts for monitoring type-I censored data using CM methodologies, focusing on keV data derived from eV measurements. The proposed control charts’ effectiveness is evaluated across various conditions, including different shift types, subgroup sizes, censoring rates, and parameter selections, comparing them to CM-EWMA and CM-CUSUM charts. Illustrating the methodology with Weibull distribution due to its relevance in reliability testing, the study also assesses the censored data charts’ performance under estimated parameters. *ARL* analysis demonstrates that CM-based DWM-EC chart outperform CM-EWMA and CM-CUSUM, attributed to the conditional median’s reduced sensitivity to extreme observations, leading to fewer false alarms. *ARL* values decrease with higher censoring rates but increase with larger shape parameter values, indicating enhanced chart efficiency. However, the performance of censored charts suffers in the estimated parameter scenario, emphasizing the importance of extensive Phase-I datasets to minimize estimation effects on chart performance. Future research could explore non-parametric control charts and investigate simultaneous estimation of shape and scale parameters’ impact.

This research introduces a novel Distance Weighted Mean based Exponentially Weighted Moving Average-Cumulative Sum control chart specifically designed to monitor keV data under type-I censoring conditions, commonly encountered in nuclear physics. By addressing the challenge of monitoring keV emissions from partially observed events and incorporating conditional median methods, the study enhances the detection of mean shifts in Weibull lifetimes with censored data. The proposed control chart’s performance is validated through simulations and a real-life dataset related to the alpha decays of the 177 Lutetium isotope, demonstrating its practical applicability. This research significantly advances statistical process control by providing a robust tool for reliability engineering and nuclear physics, where handling censored data is critical.

The limitations of the study are:

- **Type-I Censoring Focus**: The study is primarily focused on type-I censoring, which is often used in industry, even though there are other types of censoring methods such as type-II censoring that might be relevant and could provide additional insights if included.
- **Assumption of Known Scale Parameter**: The methodology fixes the shape parameter and assumes the scale parameter is known. However, in practice, these parameters often need to be estimated from the Phase-I dataset, which might introduce estimation errors.
- **Specific Distribution Assumption**: The study emphasizes monitoring the mean level of the Weibull distribution, which is useful in reliability engineering and nuclear physics. However, the methodology might not be directly applicable to other distributions of life expectancy without significant adjustments.
- **Simulation-Based Validation**: The performance of the proposed control charts is evaluated through simulations and a specific real-life dataset. While this provides some validation, broader application and testing across different scenarios and datasets would strengthen the generalizability of the findings.
- **Limited Real-Life Data**: The study discusses a real-life dataset on E(keV) related to the alpha decays of the 177 Lutetium isotope, but more diverse real-life examples could provide a more comprehensive validation of the proposed methodology.

These limitations suggest areas for future research, such as exploring other types of censoring, testing with different distributions, improving parameter estimation techniques, and validating with more diverse datasets.

## Supporting information

S1 Appendix Simulated samples used in the study. (DOCX)

S2 Appendix Data on kiloelectron-volt emission. (DOCX)
