# Supplementary material for: Investigating the average kiloelectron-volt emission of partially observed events in nuclear physics through distance weighted mean based censored control chart
Source: PLoS One. 2024 Nov 5;19(11):e0308822. doi: 10.1371/journal.pone.0308822 (PMC11537400; doi:10.1371/journal.pone.0308822)
Supplement: S1 Appendix — (DOCX) [file pone.0308822.s001.docx]

**Appendix A1 : Simulated Samples used in the study**

| Samples | | | | | Samples | | | | |
| --- | --- | --- | --- | --- | --- | --- | --- | --- | --- |
| 5.675529 | 5.10485 | 5.250828 | 5.343735667 | 5.509632333 | 4.540502 | 4.325219 | 9.057352 | 5.974357667 | 5.257429833 |
| 4.306671 | 3.911458 | 5.974296 | 4.730808333 | 4.518739667 | 3.974602 | 4.790526 | 4.620093 | 4.461740333 | 4.218171167 |
| 5.811056 | 4.351492 | 5.580828 | 5.247792 | 5.529424 | 5.57129 | 6.376151 | 6.997416 | 6.314952333 | 5.943121167 |
| 3.765854 | 5.837349 | 5.943186 | 5.182129667 | 4.473991833 | 4.099452 | 2.310166 | 5.280023 | 3.896547 | 3.9979995 |
| 4.856204 | 6.08718 | 3.066713 | 4.670032333 | 4.763118167 | 3.540908 | 7.135742 | 5.236984 | 5.304544667 | 4.422726333 |
| 2.25401 | 4.541966 | 3.959618 | 3.585198 | 2.919604 | 5.862522 | 5.468016 | 7.681953 | 6.337497 | 6.1000095 |
| 3.721541 | 5.88579 | 3.704357 | 4.437229333 | 4.079385167 | 3.398433 | 6.825256 | 4.511027 | 4.911572 | 4.1550025 |
| 7.142865 | 4.803882 | 6.118206 | 6.021651 | 6.582258 | 4.951761 | 3.419356 | 4.453616 | 4.274911 | 4.613336 |
| 5.671357 | 3.11572 | 4.863337 | 4.550138 | 5.1107475 | 3.429133 | 3.844412 | 6.047054 | 4.440199667 | 3.934666333 |
| 5.68822 | 6.833511 | 3.07235 | 5.198027 | 5.4431235 | 5.134808 | 3.881292 | 4.129734 | 4.381944667 | 4.758376333 |
| 5.428717 | 2.88539 | 5.094755 | 4.469620667 | 4.949168833 | 4.288021 | 3.249454 | 3.223976 | 3.587150333 | 3.937585667 |
| 6.021958 | 4.421367 | 4.728521 | 5.057282 | 5.53962 | 5.888044 | 2.889265 | 5.429319 | 4.735542667 | 5.311793333 |
| 3.321292 | 4.616851 | 3.226516 | 3.721553 | 3.5214225 | 4.788309 | 5.147643 | 4.329253 | 4.755068333 | 4.771688667 |
| 6.365204 | 5.873771 | 4.885739 | 5.708238 | 6.036721 | 4.715687 | 7.177859 | 4.26067 | 5.384738667 | 5.050212833 |
| 6.633603 | 6.01011 | 4.689272 | 5.777661667 | 6.205632333 | 4.624745 | 7.194517 | 5.131934 | 5.650398667 | 5.137571833 |
| 3.528413 | 6.235686 | 4.596872 | 4.786990333 | 4.157701667 | 6.943164 | 4.347986 | 3.497123 | 4.929424333 | 5.936294167 |
| 4.880359 | 3.502575 | 3.6105 | 3.997811333 | 4.439085167 | 3.429133 | 3.844412 | 6.047054 | 4.440199667 | 3.934666333 |
| 7.217269 | 4.975451 | 3.825802 | 5.339507333 | 6.278388167 | 5.134808 | 3.881292 | 4.129734 | 4.381944667 | 4.758376333 |
| 4.717599 | 6.416565 | 3.981648 | 5.038604 | 4.8781015 | 4.288021 | 3.249454 | 3.223976 | 3.587150333 | 3.937585667 |
| 2.920784 | 4.282614 | 3.016035 | 3.406477667 | 3.163630833 | 5.888044 | 2.889265 | 5.429319 | 4.735542667 | 5.311793333 |
| 4.449197 | 5.709388 | 5.451159 | 5.203248 | 4.8262225 | 4.788309 | 5.147643 | 4.329253 | 4.755068333 | 4.771688667 |
| 7.050713 | 3.364944 | 5.103587 | 5.173081333 | 6.111897167 | 4.715687 | 7.177859 | 4.26067 | 5.384738667 | 5.050212833 |
| 4.153359 | 4.565328 | 5.283764 | 4.667483667 | 4.410421333 | 7.217269 | 4.975451 | 3.825802 | 5.339507333 | 6.278388167 |
| 5.191911 | 4.482289 | 5.07797 | 4.91739 | 5.0546505 | 4.717599 | 6.416565 | 3.981648 | 5.038604 | 4.8781015 |
| 3.806668 | 4.020909 | 5.541407 | 4.456328 | 4.131498 | 2.920784 | 4.282614 | 3.016035 | 3.406477667 | 3.163630833 |
| 5.91171 | 6.822834 | 4.899645 | 5.878063 | 5.8948865 | 4.449197 | 5.709388 | 5.451159 | 5.203248 | 4.8262225 |
| 4.763376 | 2.985271 | 3.383203 | 3.710616667 | 4.236996333 | 7.050713 | 3.364944 | 5.103587 | 5.173081333 | 6.111897167 |
| 5.828828 | 4.141981 | 5.292341 | 5.087716667 | 5.458272333 | 4.153359 | 4.565328 | 5.283764 | 4.667483667 | 4.410421333 |
| 4.823521 | 5.852225 | 5.693756 | 5.456500667 | 5.140010833 | 5.191911 | 4.482289 | 5.07797 | 4.91739 | 5.0546505 |
| 4.901828 | 4.245094 | 4.67986 | 4.608927333 | 4.755377667 | 3.806668 | 4.020909 | 5.541407 | 4.456328 | 4.131498 |
| 6.534007 | 4.337895 | 6.423141 | 5.765014333 | 6.149510667 | 5.91171 | 6.822834 | 4.899645 | 5.878063 | 5.8948865 |
| 6.624266 | 3.889244 | 5.390714 | 5.301408 | 5.962837 | 4.763376 | 2.985271 | 3.383203 | 3.710616667 | 4.236996333 |
| 5.845819 | 5.827471 | 4.531777 | 5.401689 | 5.623754 | 5.828828 | 4.141981 | 5.292341 | 5.087716667 | 5.458272333 |
| 5.474306 | 4.245077 | 3.312901 | 4.344094667 | 4.909200333 | 4.823521 | 5.852225 | 5.693756 | 5.456500667 | 5.140010833 |
| 4.388281 | 4.210381 | 5.562507 | 4.720389667 | 4.554335333 | 4.901828 | 4.245094 | 4.67986 | 4.608927333 | 4.755377667 |
| 5.429981 | 4.276572 | 4.269004 | 4.658519 | 5.04425 | 6.534007 | 4.337895 | 6.423141 | 5.765014333 | 6.149510667 |
| 4.182783 | 5.702815 | 4.985842 | 4.957146667 | 4.569964833 | 6.624266 | 3.889244 | 5.390714 | 5.301408 | 5.962837 |
| 6.191727 | 3.32326 | 2.309483 | 3.94149 | 5.0666085 | 5.845819 | 5.827471 | 4.531777 | 5.401689 | 5.623754 |
| 7.340281 | 6.166309 | 2.666247 | 5.390945667 | 6.365613333 | 5.474306 | 4.245077 | 3.312901 | 4.344094667 | 4.909200333 |
| 4.783173 | 6.046561 | 5.281741 | 5.370491667 | 5.076832333 | 4.388281 | 4.210381 | 5.562507 | 4.720389667 | 4.554335333 |
| 4.424859 | 4.689727 | 4.578742 | 4.564442667 | 4.494650833 | 5.429981 | 4.276572 | 4.269004 | 4.658519 | 5.04425 |
| 7.749881 | 6.008586 | 4.253907 | 6.004124667 | 6.877002833 | 4.182783 | 5.702815 | 4.985842 | 4.957146667 | 4.569964833 |
| 7.411178 | 6.683396 | 4.334621 | 6.143065 | 6.7771215 | 6.191727 | 3.32326 | 2.309483 | 3.94149 | 5.0666085 |
| 7.725089 | 6.209489 | 4.06349 | 5.999356 | 6.8622225 | 7.340281 | 6.166309 | 2.666247 | 5.390945667 | 6.365613333 |
| 4.098049 | 6.063624 | 3.713837 | 4.62517 | 4.3616095 | 4.783173 | 6.046561 | 5.281741 | 5.370491667 | 5.076832333 |
| 5.638004 | 4.938797 | 5.641523 | 5.406108 | 5.522056 | 4.424859 | 4.689727 | 4.578742 | 4.564442667 | 4.494650833 |
| 2.261085 | 7.56727 | 4.174903 | 4.667752667 | 3.464418833 | 7.749881 | 6.008586 | 4.253907 | 6.004124667 | 6.877002833 |
| 4.739951 | 5.920966 | 4.13286 | 4.931259 | 4.835605 | 7.411178 | 6.683396 | 4.334621 | 6.143065 | 6.7771215 |
| 4.48649 | 5.783055 | 4.679288 | 4.982944333 | 4.734717167 | 7.725089 | 6.209489 | 4.06349 | 5.999356 | 6.8622225 |
| 5.106262 | 2.87988 | 4.260907 | 4.082349667 | 4.594305833 | 4.098049 | 6.063624 | 3.713837 | 4.62517 | 4.3616095 |
| 5.294058 | 3.442415 | 4.944924 | 4.560465667 | 4.927261833 | 5.638004 | 4.938797 | 5.641523 | 5.406108 | 5.522056 |
| 4.540502 | 4.325219 | 9.057352 | 5.974357667 | 5.257429833 | 2.261085 | 7.56727 | 4.174903 | 4.667752667 | 3.464418833 |
| 3.974602 | 4.790526 | 4.620093 | 4.461740333 | 4.218171167 | 4.739951 | 5.920966 | 4.13286 | 4.931259 | 4.835605 |
| 5.57129 | 6.376151 | 6.997416 | 6.314952333 | 5.943121167 | 4.48649 | 5.783055 | 4.679288 | 4.982944333 | 4.734717167 |
| 4.099452 | 2.310166 | 5.280023 | 3.896547 | 3.9979995 | 5.106262 | 2.87988 | 4.260907 | 4.082349667 | 4.594305833 |
| 3.540908 | 7.135742 | 5.236984 | 5.304544667 | 4.422726333 | 5.294058 | 3.442415 | 4.944924 | 4.560465667 | 4.927261833 |
| 5.862522 | 5.468016 | 7.681953 | 6.337497 | 6.1000095 | 4.540502 | 4.325219 | 9.057352 | 5.974357667 | 5.257429833 |
| 3.398433 | 6.825256 | 4.511027 | 4.911572 | 4.1550025 | 3.974602 | 4.790526 | 4.620093 | 4.461740333 | 4.218171167 |
| 4.951761 | 3.419356 | 4.453616 | 4.274911 | 4.613336 | 5.57129 | 6.376151 | 6.997416 | 6.314952333 | 5.943121167 |
| 3.429133 | 3.844412 | 6.047054 | 4.440199667 | 3.934666333 | 4.099452 | 2.310166 | 5.280023 | 3.896547 | 3.9979995 |
| 5.134808 | 3.881292 | 4.129734 | 4.381944667 | 4.758376333 | 3.540908 | 7.135742 | 5.236984 | 5.304544667 | 4.422726333 |
| 4.288021 | 3.249454 | 3.223976 | 3.587150333 | 3.937585667 | 5.862522 | 5.468016 | 7.681953 | 6.337497 | 6.1000095 |
| 5.888044 | 2.889265 | 5.429319 | 4.735542667 | 5.311793333 | 3.398433 | 6.825256 | 4.511027 | 4.911572 | 4.1550025 |
| 4.788309 | 5.147643 | 4.329253 | 4.755068333 | 4.771688667 | 4.951761 | 3.419356 | 4.453616 | 4.274911 | 4.613336 |
| 4.715687 | 7.177859 | 4.26067 | 5.384738667 | 5.050212833 | 3.429133 | 3.844412 | 6.047054 | 4.440199667 | 3.934666333 |
| 4.624745 | 7.194517 | 5.131934 | 5.650398667 | 5.137571833 | 5.134808 | 3.881292 | 4.129734 | 4.381944667 | 4.758376333 |
| 6.943164 | 4.347986 | 3.497123 | 4.929424333 | 5.936294167 | 4.288021 | 3.249454 | 3.223976 | 3.587150333 | 3.937585667 |
| 4.98238 | 5.255177 | 7.038943 | 5.758833333 | 5.370606667 | 5.888044 | 2.889265 | 5.429319 | 4.735542667 | 5.311793333 |
| 6.785792 | 7.407878 | 5.563556 | 6.585742 | 6.685767 | 4.788309 | 5.147643 | 4.329253 | 4.755068333 | 4.771688667 |
| 3.321292 | 4.616851 | 3.226516 | 3.721553 | 3.5214225 | 4.715687 | 7.177859 | 4.26067 | 5.384738667 | 5.050212833 |
| 6.365204 | 5.873771 | 4.885739 | 5.708238 | 6.036721 | 4.624745 | 7.194517 | 5.131934 | 5.650398667 | 5.137571833 |
| 6.633603 | 6.01011 | 4.689272 | 5.777661667 | 6.205632333 | 6.943164 | 4.347986 | 3.497123 | 4.929424333 | 5.936294167 |
| 3.528413 | 6.235686 | 4.596872 | 4.786990333 | 4.157701667 | 4.98238 | 5.255177 | 7.038943 | 5.758833333 | 5.370606667 |
| 4.880359 | 3.502575 | 3.6105 | 3.997811333 | 4.439085167 | 6.785792 | 7.407878 | 5.563556 | 6.585742 | 6.685767 |
| 7.217269 | 4.975451 | 3.825802 | 5.339507333 | 6.278388167 | 5.106262 | 2.87988 | 4.260907 | 4.082349667 | 4.594305833 |
| 4.717599 | 6.416565 | 3.981648 | 5.038604 | 4.8781015 | 5.294058 | 3.442415 | 4.944924 | 4.560465667 | 4.927261833 |
| 2.920784 | 4.282614 | 3.016035 | 3.406477667 | 3.163630833 | 4.540502 | 4.325219 | 9.057352 | 5.974357667 | 5.257429833 |
| 4.449197 | 5.709388 | 5.451159 | 5.203248 | 4.8262225 | 3.974602 | 4.790526 | 4.620093 | 4.461740333 | 4.218171167 |
| 7.050713 | 3.364944 | 5.103587 | 5.173081333 | 6.111897167 | 5.57129 | 6.376151 | 6.997416 | 6.314952333 | 5.943121167 |
| 4.153359 | 4.565328 | 5.283764 | 4.667483667 | 4.410421333 | 4.099452 | 2.310166 | 5.280023 | 3.896547 | 3.9979995 |
| 5.191911 | 4.482289 | 5.07797 | 4.91739 | 5.0546505 | 3.540908 | 7.135742 | 5.236984 | 5.304544667 | 4.422726333 |
| 3.806668 | 4.020909 | 5.541407 | 4.456328 | 4.131498 | 5.862522 | 5.468016 | 7.681953 | 6.337497 | 6.1000095 |
| 5.91171 | 6.822834 | 4.899645 | 5.878063 | 5.8948865 | 3.398433 | 6.825256 | 4.511027 | 4.911572 | 4.1550025 |
| 4.763376 | 2.985271 | 3.383203 | 3.710616667 | 4.236996333 | 4.951761 | 3.419356 | 4.453616 | 4.274911 | 4.613336 |
| 5.828828 | 4.141981 | 5.292341 | 5.087716667 | 5.458272333 | 3.429133 | 3.844412 | 6.047054 | 4.440199667 | 3.934666333 |
| 4.823521 | 5.852225 | 5.693756 | 5.456500667 | 5.140010833 | 5.134808 | 3.881292 | 4.129734 | 4.381944667 | 4.758376333 |
| 4.901828 | 4.245094 | 4.67986 | 4.608927333 | 4.755377667 | 4.288021 | 3.249454 | 3.223976 | 3.587150333 | 3.937585667 |
| 6.534007 | 4.337895 | 6.423141 | 5.765014333 | 6.149510667 | 5.888044 | 2.889265 | 5.429319 | 4.735542667 | 5.311793333 |
| 6.624266 | 3.889244 | 5.390714 | 5.301408 | 5.962837 | 4.788309 | 5.147643 | 4.329253 | 4.755068333 | 4.771688667 |
| 5.845819 | 5.827471 | 4.531777 | 5.401689 | 5.623754 | 4.715687 | 7.177859 | 4.26067 | 5.384738667 | 5.050212833 |
| 5.474306 | 4.245077 | 3.312901 | 4.344094667 | 4.909200333 | 4.624745 | 7.194517 | 5.131934 | 5.650398667 | 5.137571833 |
| 4.388281 | 4.210381 | 5.562507 | 4.720389667 | 4.554335333 | 6.943164 | 4.347986 | 3.497123 | 4.929424333 | 5.936294167 |
| 5.429981 | 4.276572 | 4.269004 | 4.658519 | 5.04425 | 3.429133 | 3.844412 | 6.047054 | 4.440199667 | 3.934666333 |
| 4.182783 | 5.702815 | 4.985842 | 4.957146667 | 4.569964833 | 5.134808 | 3.881292 | 4.129734 | 4.381944667 | 4.758376333 |
| 6.191727 | 3.32326 | 2.309483 | 3.94149 | 5.0666085 | 4.288021 | 3.249454 | 3.223976 | 3.587150333 | 3.937585667 |
| 7.340281 | 6.166309 | 2.666247 | 5.390945667 | 6.365613333 | 5.888044 | 2.889265 | 5.429319 | 4.735542667 | 5.311793333 |
| 4.783173 | 6.046561 | 5.281741 | 5.370491667 | 5.076832333 | 4.788309 | 5.147643 | 4.329253 | 4.755068333 | 4.771688667 |
| 4.424859 | 4.689727 | 4.578742 | 4.564442667 | 4.494650833 | 2.261085 | 7.56727 | 4.174903 | 4.667752667 | 3.464418833 |
| 7.749881 | 6.008586 | 4.253907 | 6.004124667 | 6.877002833 | 4.739951 | 5.920966 | 4.13286 | 4.931259 | 4.835605 |
| 7.411178 | 6.683396 | 4.334621 | 6.143065 | 6.7771215 | 4.48649 | 5.783055 | 4.679288 | 4.982944333 | 4.734717167 |
| 7.725089 | 6.209489 | 4.06349 | 5.999356 | 6.8622225 | 5.106262 | 2.87988 | 4.260907 | 4.082349667 | 4.594305833 |
| 4.098049 | 6.063624 | 3.713837 | 4.62517 | 4.3616095 | 5.294058 | 3.442415 | 4.944924 | 4.560465667 | 4.927261833 |
| 5.638004 | 4.938797 | 5.641523 | 5.406108 | 5.522056 | 4.540502 | 4.325219 | 9.057352 | 5.974357667 | 5.257429833 |
| 2.261085 | 7.56727 | 4.174903 | 4.667752667 | 3.464418833 | 3.974602 | 4.790526 | 4.620093 | 4.461740333 | 4.218171167 |
| 4.739951 | 5.920966 | 4.13286 | 4.931259 | 4.835605 | 5.57129 | 6.376151 | 6.997416 | 6.314952333 | 5.943121167 |
| 4.48649 | 5.783055 | 4.679288 | 4.982944333 | 4.734717167 | 4.099452 | 2.310166 | 5.280023 | 3.896547 | 3.9979995 |
| 5.106262 | 2.87988 | 4.260907 | 4.082349667 | 4.594305833 | 3.540908 | 7.135742 | 5.236984 | 5.304544667 | 4.422726333 |
| 5.294058 | 3.442415 | 4.944924 | 4.560465667 | 4.927261833 | 5.862522 | 5.468016 | 7.681953 | 6.337497 | 6.1000095 |
| 4.540502 | 4.325219 | 9.057352 | 5.974357667 | 5.257429833 | 3.398433 | 6.825256 | 4.511027 | 4.911572 | 4.1550025 |
| 3.974602 | 4.790526 | 4.620093 | 4.461740333 | 4.218171167 | 4.951761 | 3.419356 | 4.453616 | 4.274911 | 4.613336 |
| 5.57129 | 6.376151 | 6.997416 | 6.314952333 | 5.943121167 | 3.429133 | 3.844412 | 6.047054 | 4.440199667 | 3.934666333 |
| 4.099452 | 2.310166 | 5.280023 | 3.896547 | 3.9979995 | 5.134808 | 3.881292 | 4.129734 | 4.381944667 | 4.758376333 |
| 3.540908 | 7.135742 | 5.236984 | 5.304544667 | 4.422726333 | 4.288021 | 3.249454 | 3.223976 | 3.587150333 | 3.937585667 |
| 5.862522 | 5.468016 | 7.681953 | 6.337497 | 6.1000095 | 5.888044 | 2.889265 | 5.429319 | 4.735542667 | 5.311793333 |
| 3.398433 | 6.825256 | 4.511027 | 4.911572 | 4.1550025 | 4.788309 | 5.147643 | 4.329253 | 4.755068333 | 4.771688667 |
| 4.951761 | 3.419356 | 4.453616 | 4.274911 | 4.613336 | 4.715687 | 7.177859 | 4.26067 | 5.384738667 | 5.050212833 |
| 3.429133 | 3.844412 | 6.047054 | 4.440199667 | 3.934666333 | 4.624745 | 7.194517 | 5.131934 | 5.650398667 | 5.137571833 |
| 5.134808 | 3.881292 | 4.129734 | 4.381944667 | 4.758376333 | 6.943164 | 4.347986 | 3.497123 | 4.929424333 | 5.936294167 |
| 4.288021 | 3.249454 | 3.223976 | 3.587150333 | 3.937585667 | 4.98238 | 5.255177 | 7.038943 | 5.758833333 | 5.370606667 |
| 5.888044 | 2.889265 | 5.429319 | 4.735542667 | 5.311793333 | 6.785792 | 7.407878 | 5.563556 | 6.585742 | 6.685767 |
| 4.788309 | 5.147643 | 4.329253 | 4.755068333 | 4.771688667 | 5.106262 | 2.87988 | 4.260907 | 4.082349667 | 4.594305833 |
| 4.715687 | 7.177859 | 4.26067 | 5.384738667 | 5.050212833 | 5.294058 | 3.442415 | 4.944924 | 4.560465667 | 4.927261833 |
| 4.624745 | 7.194517 | 5.131934 | 5.650398667 | 5.137571833 | 4.540502 | 4.325219 | 9.057352 | 5.974357667 | 5.257429833 |
| 6.943164 | 4.347986 | 3.497123 | 4.929424333 | 5.936294167 | 3.974602 | 4.790526 | 4.620093 | 4.461740333 | 4.218171167 |
| 4.98238 | 5.255177 | 7.038943 | 5.758833333 | 5.370606667 | 5.57129 | 6.376151 | 6.997416 | 6.314952333 | 5.943121167 |
| 6.785792 | 7.407878 | 5.563556 | 6.585742 | 6.685767 | 4.099452 | 2.310166 | 5.280023 | 3.896547 | 3.9979995 |
| 5.106262 | 2.87988 | 4.260907 | 4.082349667 | 4.594305833 | 3.540908 | 7.135742 | 5.236984 | 5.304544667 | 4.422726333 |
| 5.294058 | 3.442415 | 4.944924 | 4.560465667 | 4.927261833 | 5.862522 | 5.468016 | 7.681953 | 6.337497 | 6.1000095 |
| 5.693756 | 5.456500667 | 5.140010833 | 5.191911 | 4.482289 | 3.398433 | 6.825256 | 4.511027 | 4.911572 | 4.1550025 |
| 4.67986 | 4.608927333 | 4.755377667 | 3.806668 | 4.020909 | 4.951761 | 3.419356 | 4.453616 | 4.274911 | 4.613336 |
| 6.423141 | 5.765014333 | 6.149510667 | 5.91171 | 6.822834 | 3.429133 | 3.844412 | 6.047054 | 4.440199667 | 3.934666333 |
| 5.390714 | 5.301408 | 5.962837 | 4.763376 | 2.985271 | 4.131498 | 5.862522 | 5.468016 | 7.681953 | 6.337497 |
| 4.531777 | 5.401689 | 5.623754 | 5.828828 | 4.141981 | 5.8948865 | 3.398433 | 6.825256 | 4.511027 | 4.911572 |
| 3.312901 | 4.344094667 | 4.909200333 | 4.823521 | 5.852225 | 4.236996333 | 4.951761 | 3.419356 | 4.453616 | 4.274911 |
| 5.562507 | 4.720389667 | 4.554335333 | 4.901828 | 4.245094 | 5.458272333 | 3.429133 | 3.844412 | 6.047054 | 4.440199667 |
| 4.269004 | 4.658519 | 5.04425 | 6.534007 | 4.337895 | 5.140010833 | 5.134808 | 3.881292 | 4.129734 | 4.381944667 |
| 4.985842 | 4.957146667 | 4.569964833 | 6.624266 | 3.889244 | 4.755377667 | 4.288021 | 3.249454 | 3.223976 | 3.587150333 |
| 2.309483 | 3.94149 | 5.0666085 | 5.845819 | 5.827471 | 6.149510667 | 5.888044 | 2.889265 | 5.429319 | 4.735542667 |
| 2.666247 | 5.390945667 | 6.365613333 | 5.474306 | 4.245077 | 5.962837 | 4.788309 | 5.147643 | 4.329253 | 4.755068333 |
| 5.281741 | 5.370491667 | 5.076832333 | 4.388281 | 4.210381 | 5.623754 | 4.715687 | 7.177859 | 4.26067 | 5.384738667 |
| 4.578742 | 4.564442667 | 4.494650833 | 5.429981 | 4.276572 | 4.909200333 | 4.624745 | 7.194517 | 5.131934 | 5.650398667 |
| 4.253907 | 6.004124667 | 6.877002833 | 4.182783 | 5.702815 | 4.554335333 | 6.943164 | 4.347986 | 3.497123 | 4.929424333 |
| 4.334621 | 6.143065 | 6.7771215 | 6.191727 | 3.32326 | 5.04425 | 3.429133 | 3.844412 | 6.047054 | 4.440199667 |
| 4.06349 | 5.999356 | 6.8622225 | 7.340281 | 6.166309 | 4.569964833 | 5.134808 | 3.881292 | 4.129734 | 4.381944667 |
| 3.713837 | 4.62517 | 4.3616095 | 4.783173 | 6.046561 | 5.0666085 | 4.288021 | 3.249454 | 3.223976 | 3.587150333 |
| 5.641523 | 5.406108 | 5.522056 | 4.424859 | 4.689727 | 6.365613333 | 5.888044 | 2.889265 | 5.429319 | 4.735542667 |
| 4.174903 | 4.667752667 | 3.464418833 | 7.749881 | 6.008586 | 5.076832333 | 4.788309 | 5.147643 | 4.329253 | 4.755068333 |
| 4.13286 | 4.931259 | 4.835605 | 7.411178 | 6.683396 | 4.494650833 | 2.261085 | 7.56727 | 4.174903 | 4.667752667 |
| 4.679288 | 4.982944333 | 4.734717167 | 7.725089 | 6.209489 | 6.877002833 | 4.739951 | 5.920966 | 4.13286 | 4.931259 |
| 4.260907 | 4.082349667 | 4.594305833 | 4.098049 | 6.063624 | 6.7771215 | 4.48649 | 5.783055 | 4.679288 | 4.982944333 |
| 4.944924 | 4.560465667 | 4.927261833 | 5.638004 | 4.938797 | 6.8622225 | 5.106262 | 2.87988 | 4.260907 | 4.082349667 |
| 9.057352 | 5.974357667 | 5.257429833 | 2.261085 | 7.56727 | 4.3616095 | 5.294058 | 3.442415 | 4.944924 | 4.560465667 |
| 4.620093 | 4.461740333 | 4.218171167 | 4.739951 | 5.920966 | 5.522056 | 4.540502 | 4.325219 | 9.057352 | 5.974357667 |
| 6.997416 | 6.314952333 | 5.943121167 | 4.48649 | 5.783055 | 3.464418833 | 3.974602 | 4.790526 | 4.620093 | 4.461740333 |
| 5.280023 | 3.896547 | 3.9979995 | 5.106262 | 2.87988 | 4.835605 | 5.57129 | 6.376151 | 6.997416 | 6.314952333 |
| 5.236984 | 5.304544667 | 4.422726333 | 5.294058 | 3.442415 | 4.734717167 | 4.099452 | 2.310166 | 5.280023 | 3.896547 |
| 7.681953 | 6.337497 | 6.1000095 | 4.540502 | 4.325219 | 4.594305833 | 3.540908 | 7.135742 | 5.236984 | 5.304544667 |
| 4.511027 | 4.911572 | 4.1550025 | 3.974602 | 4.790526 | 4.927261833 | 5.862522 | 5.468016 | 7.681953 | 6.337497 |
| 4.453616 | 4.274911 | 4.613336 | 5.57129 | 6.376151 | 5.257429833 | 3.398433 | 6.825256 | 4.511027 | 4.911572 |
| 6.047054 | 4.440199667 | 3.934666333 | 4.099452 | 2.310166 | 4.218171167 | 4.951761 | 3.419356 | 4.453616 | 4.274911 |
| 4.129734 | 4.381944667 | 4.758376333 | 3.540908 | 7.135742 | 5.943121167 | 3.429133 | 3.844412 | 6.047054 | 4.440199667 |
| 3.223976 | 3.587150333 | 3.937585667 | 5.862522 | 5.468016 | 3.9979995 | 5.134808 | 3.881292 | 4.129734 | 4.381944667 |
| 5.429319 | 4.735542667 | 5.311793333 | 3.398433 | 6.825256 | 4.422726333 | 4.288021 | 3.249454 | 3.223976 | 3.587150333 |
