# Supplementary material for: Investigating the average kiloelectron-volt emission of partially observed events in nuclear physics through distance weighted mean based censored control chart
Source: PLoS One. 2024 Nov 5;19(11):e0308822. doi: 10.1371/journal.pone.0308822 (PMC11537400; doi:10.1371/journal.pone.0308822)
Supplement: S2 Appendix — (DOCX) [file pone.0308822.s002.docx]

**Appendix A2: Data on Kiloelectron-Volt Emission**

| **X-rays from ^177^Lu (6.734 d *12*)**  (**E (keV) for** Censored observation-(C)) | | |
| --- | --- | --- |
| **E (keV)** | **I (%)** | **Assignment** |
|  | | |
| 6.960 | 0.053 *6* | Hf L*_l_* |
| 7.844 (C) | 0.129 *9* | Hf L_a2_ |
| 7.899 | 1.15 *8* | Hf L_a1_ |
| 8.139(C) | 0.0227 *16* | Hf L_h_ |
| 8.905(C) | 0.031 *5* | Hf L_b4_ |
| 9.023 | 0.0139 *10* | Hf L_b6_ |
| 9.023 | 1.09 *8* | Hf L_b1_ |
| 9.163 | 0.046 *7* | Hf L_b3_ |
| 9.342(C) | 0.245 *18* | Hf L_b2_ |
| 9.554 | 0.00345 *25* | Hf L_b5_ |
| 10.516 | 0.201 *15* | Hf L_g1_ |
| 10.733(C) | 0.0031 *3* | Hf L_g6_ |
| 10.834 | 0.0099 *17* | Hf L_g2_ |
| 10.890 | 0.0143 *24* | Hf L_g3_ |
| 54.080 | 0.00102 *5* | Hf K_a3_ |
| 54.611 | 1.62 *8* | Hf K_a2_ |
| 55.790 | 2.83 *13* | Hf K_a1_ |
| 62.985 | 0.311 *15* | Hf K_b3_ |
| 63.243 | 0.60 *3* | Hf K_b1_ |
| 63.662 | 0.0131 *8* | Hf K_b5_ |
| 64.942 | 0.206 *10* | Hf K_b2_ |
| 65.132 | 0.0354 *21* | Hf K_b4_ |

**(Data retrived from: https://nucleardata.nuclear.lu.se/toi/nuclide.asp?iZA=710177&sortG=E&sortA=E)**
